# Bilberry Expansion in the Changing Subalpine Belt

**DOI:** 10.3390/plants13182633

**Published:** 2024-09-20

**Authors:** Miroslav Zeidler, Marek Banaš

**Affiliations:** Department of Ecology and Environmental Sciences, Faculty of Science, Palacký University Olomouc, 779 00 Olomouc, Czech Republic

**Keywords:** shrubification bilberry, *Vaccinium myrtillus*, subalpine, mountains, plant traits, habitat

## Abstract

Bilberry (*Vaccinium myrtillus* L.) expansion in subalpine and alpine ecosystems is increasing due to climate change and reduced land management. This review examines bilberry traits, environmental responses, and ecosystem impacts. As a stress-tolerant chamaephyte, bilberry thrives in acidic, nutrient-poor soils across various habitats. It propagates effectively through rhizomes and demonstrates a phalanx growth form. Bilberry’s growth and distribution are influenced by elevation, soil structure, pH, water availability, and nitrogen content. Mycorrhizal associations play a crucial role in nutrient uptake. The species modifies the microclimate, facilitates litter accumulation, and influences soil microbial communities, affecting nutrient turnover and biodiversity. Bilberry shows moderate tolerance to herbivory and frost, with the ability to recover through rapid emergence of new ramets. However, severe or repeated disturbances can significantly impact its abundance and reproductive success. Climate warming and atmospheric nitrogen deposition have accelerated bilberry growth in treeline ecotones. The management of bilberry expansion requires a nuanced approach, considering its resilience, historical land-use changes, and environmental factors. The goal should be to limit, not eliminate, bilberry, as it is a natural part of subalpine communities. Long-term comparative monitoring and experimental manipulation are necessary for effective management strategies.

## 1. Introduction

Increasing shrub dominance at northern or upper elevational range edges, both in the tundra biome [1,2] and alpine belt worldwide [3,4], is attributed to rapid climate change and human impact [5,6]. Deciduous shrub expansion has been particularly linked to accelerating air temperature rise, enhanced soil nutrient mineralization, and other accompanying factors, including their interactions [7,8,9,10,11,12].

An increasing trend in annual growth increment has been documented in the Alps since the 1990s based on bilberry growth ring data [13]. However, woody shrub encroachment has been regionally observed since the 1950s and for other shrubby species worldwide since the 1970s [13,14]. Rapid expansion rates of 0.6–16% per year have been recorded in the subalpine belt of the High Sudetes Mts. [14] and 5.6% per decade at altitudes between 2400 m a.s.l. and 2500 m a.s.l. in the European Alps [8].

Shrub traits, such as shoot length, leaf number, abundance, and biomass, are commonly found to be sensitive indicators of environmental change and ecosystem functioning [1,5,13,15]. Conversely, shrub encroachment can modify biotic and abiotic environmental conditions, significantly causing vegetation shifts, altering ecosystem functioning and diversity, and potentially modifying the climate by changing surface albedo, energy and water balance, and permafrost [8,16,17,18]. However, a comprehensive view of the shrub encroachment and its consequences remain poorly synthesized in detail.

Recent documentation of shrubification has focused on *Alnus viridis* [19], *Juniperus communis* L. [20], *Rhododendron ferrugineum* [21], and *Pinus mugo* [4,14] in European mountain regions. Understanding both the aboveground and belowground effects of woody encroachment is crucial for predicting future changes in alpine ecosystem structure and function as well as subsequent feedback to the global climate system [18]. The impacts of shrub encroachment on ecosystem structure and functioning, whether positive or negative, are heavily dependent on the functional traits of both the encroaching shrubs and the plant communities they replace [17]. This review focuses specifically on the common species, bilberry (*Vaccinium myrtillus* L.), as only a limited number of shrub species (chamaephytes) have a sufficiently large distribution area to assess their response over a broad range [13].

In the subalpine belt, bilberry and its expansion play a substantial role in the treeline ecotone and subalpine communities across major mountain ranges, such as the Pyrenees [22], the European Alps [3,4,13], and the Carpathians [23,24]. The shrub expansion is also documented in mountains of limited range and narrow alpine/subalpine belts, including the Scandes [25], the Vosges and the Massif Central [26], the Scottish uplands [27], and the High Sudetes [28,29].

At subalpine and alpine belts, climate warming is likely to lead to milder winters and/or earlier springs. Warmer winters can cause premature snowmelt, leading to early dehardening in plants or the prevention of rehardening, especially if diurnal temperatures are already high. In the 1990s, even dieback occurrence was predicted for bilberry [30,31]. Delayed metabolic activity, retarded bud burst, and reduced stem elongation in bilberry plants were anticipated as consequences of climate warming [32]. However, we now observe the expansion of shrubs, including bilberry, in forest, alpine, and tundra habitats worldwide. The following text summarizes the most important prerequisites for the spread of blueberry and its consequences.

## 2. Species Traits

Bilberry (*Vaccinium myrtillus*), a member of the Ericaceae family, is a small shrub that is typically (5-)35–60 cm tall, though its morphological parameters can vary within a single clone depending on environmental conditions [33]. Plant height and stem rigidity decrease with altitude, potentially limiting its reproductive capacity at higher elevations with strong winds [27]. Bilberry adopts a more prostrate form at higher altitudes, demonstrating the mountain environment’s effect on plant morphology. This trait persists in lowland cultivation, indicating genetic control [27].

The dwarf shrub, including *Vaccinium myrtillus* subsp. *compactum* [34], is one of the most frequent and abundant vascular plant species in northern Europe and high-altitude European mountains, thriving in subarctic and subalpine communities [33,35]. Its distribution extends across northern Asia (Siberia), Japan, and Greenland [34,36]. The species’ extensive range, from Mongolia through the Caucasus to southern European mountains, illustrates its broad ecological niche, occupying diverse habitats, from mountain forests to alpine heaths [37,38].

The deciduous chamaephyte exhibits a competitor-stress tolerator strategy typical of boreal and temperate zones [33,39,40]. It prefers acidic, nutrient-poor soils with high C/N ratios in understory and is also codominant in some open habitats, including coniferous forests, heathlands, and alpine grasslands [36,37,38]. In the alpine zone, it can reach altitudes over 2500 m [8] and colonize anthropogenic habitats like ski slopes [29]. The shrub often forms pure stands or mixes with other species such as *Vaccinium uliginosum* or *Rhododendron ferrugineum* [22]. In the ecotone of the treeline and (sub)alpine belt, it belongs to the dominants of association, *Genisto pilosae-Vaccinion*, in central Europe [41].

Bilberry tolerates both shade and full sunlight [33,42], though dense older forests are less favorable, resulting in slower growth [43]. In mountainous areas, it often forms monotonous cover on leeward slopes, especially in treeline ecotones and cirque peripheries, where snow cover melts late in spring [44,45]. The species prefers sheltered habitats on stony, shallow soils with surface raw humus accumulation [37]. As its flexible stems metamorphose into a dense network of subterranean rhizomes, bilberry spreads effectively through underground woody stems and bud banks [43]. Its roots typically reach 15–20 cm depths, spreading radially at 5–10 cm per year [33,46]. New shoot growth begins in April, while root growth starts in late May, independent of nitrogen supply [47]. The main nitrogen storage compounds in rhizomes and older stems include arginine, urea, glutamine, and various ammonium compounds. Both arginine and glutamine serve as important mobilizable and translocable nitrogen storage compounds in bilberry plants [48]. The species forms dense, expanding cover that accumulates raw humus beneath [46].

This chamaephyte is adapted to snow protection, with its phenology, growth, and persistence closely linked to snow cover changes [29,49]. However, bilberry demonstrates a broad ecological tolerance for zonal temperature, habitat irradiance, and soil hydrologic and trophic regimes, as evidenced by its presence in various forest types and high-mountain tundra in the Altai Mountains [34].

## 3. Growth and Propagation

Bilberry exhibits a phalanx growth form, producing short internodes that result in an advancing front of closely packed ramets [50]. Young rhizomes grow closer to the surface than older ones, contributing to the formation of a raw humus layer. This growth pattern allows the plant to colonize and increase in density, with the centrifugal development of patches being driven by the independent growth of these small functional units [51]. The plant’s clonal growth enables it to compensate for lost biomass by activating dormant buds, even within a single season. Additionally, there is a relationship between the carbon-to-nitrogen ratio in the soil organic layer and how biomass is partitioned among different parts of the bilberry plant [52]. Molecular analysis places bilberry closer to the phalanx end of the continuum between typical phalanx and guerilla growth strategies [50]. The population is clonally structured, with clones forming discrete patches. The genotypic diversity and evenness observed in bilberry is similar to other Ericaceae species. Despite intra-population variability in clonal diversity and spatial structure, no significant differences were detected between various habitats studied in Belgium [50]. The presence of small clones isolated within larger clones supports the “recruitment at windows of opportunity” theory, where recruitment within established conspecific adult stands is limited spatially and temporally.

Bilberry suffers from inbreeding depression. The main pollinators, bumblebee queens, favor geitonogamy due to their short successive visits to flowers [53]. However, the risk of individual extinction and fitness costs of geitonogamy are compensated by advantages of clonal growth, such as increased resource acquisition and storage.

Seed germination rates in the field are typically very low [47]. However, high seed germination rates have been observed above bilberry altitudinal distribution in moist subalpine mountain habitats, indicating the potential for upward movement under a warming climate [54]. Drought, however, limits seedling recruitment at tundra sites, suggesting that warming temperatures alone may not necessarily cause a latitudinal shift. The seed bank of bilberry varies, with an average of 0–166 seeds/m^2^ in subalpine forests and conifer hardwoods, respectively [55]. Mean seed longevity is assessed at 8.65 years, with climatic factors, particularly the annual temperature range, accounting for 42% of the variation in seed longevity.

Neither flower nor fruit density varies significantly with elevation [56]. Both climatic conditions and soil nutrient availability have been proposed as principal factors affecting flower and fruit numbers [56]. Fertilized, non-clipped plants produce the heaviest fruits, while clipping alone has no significant effect on fruit mass [57]. Bilberry is capable of self-pollination, but cross-pollination by insects is crucial for seed production. This is particularly evident in alpine tundra, where pollinator exclusion resulted in an 84% lower fruit number, a 50% lower fruit mass, and a 50% lower seed mass [58]. Despite this, shrubs appear relatively robust to changes in the pollinator community in a warmer climate [58].

The timing of phenological events of bilberry is strongly influenced by environmental factors. Bud burst and growth initiation are triggered by rising temperatures after snowmelt [59]. Floral initiation occurs during a short period in mid-June, and flower primordia are formed by October, allowing for plants to develop flowers without chilling [59]. In Finnish Lapland, spring phenophases have advanced by 1–2 days/year for most studied species, including bilberry, resulting in a lengthened growth period. Autumn phenophases, however, showed no significant trends or relationships with climatic conditions [60]. The majority of mountain plants flower after the longest day of the year, suggesting that the time span for reproduction may be extended under global warming [61]. However, recovery from injuries depends on the phenological stage [62,63]. If resources are used for growth due to early snowmelt and high temperatures, the recovery ability following frost damage may be reduced [60].

## 4. Habitat Characteristics

### 4.1. Elevation

Elevation gradients are useful for examining plant responses to environmental changes, as they integrate multiple abiotic factors, though this integration can also be a drawback [64]. In temperate seasonal zones, atmospheric pressure, CO_2_ concentration, temperature, the length of the vegetation period, and nutrient availability typically decrease with increasing elevation. Conversely, annual precipitation, frost frequency during the growing season, and solar radiation tend to increase [49,64].

Bilberry growth traits decrease with increasing elevation; ramets are shorter and younger, and growth rings are thinner at higher elevations [16]. Elevation and decreasing temperature directly affect shrub cover, reducing the overall cover of this life-form [16]. Variables related to plant performance, herbivory, and fruit production are significantly affected by habitat and altitude [65]. Where competition for irradiance is not high but the growing season is short, a short, weak stem can lead to greater rates of photosynthesis than in lowlands. This is achieved through the nitrogen economy of the leaves and maximization of stomatal conductance, albeit at the expense of reproductive capacity [27]. The proportion of nitrogen in leaves, relative to the total in the stem plus leaves, increases with altitude, providing evidence for the maximization of leaf nitrogen with altitude. However, stem nitrogen content remains unchanged between altitudes of 200–1000 m, with a mean content of 0.97% [27]. The proportion of aboveground dry weight in leaves also increases with altitude in a similar ratio to the proportion of nitrogen in leaves. This suggests that reducing stem stature with altitude increases the available nitrogen supply to leaves [27].

### 4.2. Soil

Soil structure and composition is crucial for bilberry growth. Bilberry roots are mostly concentrated in mineral horizons [46], usually on the organic top horizon (O), but eluvial (E) and subsoil (B) horizons may contribute significantly to the nutrient supply [66]. The distribution of soils is affected by snow accumulation due to processes of nivation [67]. A high sand and gravel content negatively affects growth in dry periods due to reduced water holding capacity [66].

The content of plant-available P, K, Mg, and Ca is generally low in mineral soil layers. The pattern of nutrient release from decomposing bilberry leaf litter differs somewhat from site to site, with variations noted for P, Ca, Mg, N, and K [68]. However, mycorrhizal associations enhance the availability of nutrients, especially phosphorus, in these acidic soils [69]. The symbiotic relationship with ericoid mycorrhiza is crucial for bilberry, providing essential minerals for growth. This symbiosis is particularly advantageous in environments with uneven nutrient distribution and limited availability (Pladias Database of the Czech Flora and Vegetation, www.pladias.cz, accessed on 26 May 2024). Ericaceous shrub expansion, including bilberry, shifts soil fungal communities towards ericoid mycorrhizal fungi and saprotrophs associated with nutrient-poor substrates. These fungi, resistant to decay due to their melanized hyphae, may immobilize soil nutrients for extended periods, potentially leading to lower soil N availability [69]. The expansion also alters bacterial communities, favoring taxa associated with cellulose and xylan degradation. This shift is connected with increased cellulolytic enzyme activities. Bacterial communities trend towards K-selected growth strategies, specifically an increased Gram-positive-to-Gram-negative bacterial ratio, which correlates with lower soil nutrient availability and more recalcitrant C compounds in soil [69].

As a calciphobic species, bilberry thrives in substrates with pH values below 4.5 but struggles in environments with a pH above 6. The soil reaction directly affects chlorophyll content and growth of bilberry [56]. Interestingly, chlorophyll content and growth increase with elevation, peaking at 950 m, before decreasing, with both variables negatively influenced by a high soil pH [56]. Bilberry is sensitive to high total salt concentration; thus, the substrate must not dry out excessively to prevent increased salt concentration in soil water [70].

### 4.3. Water Supply

Alpine systems are projected to receive reduced precipitation [71], which makes water crucial not only for bilberry. Analyses on *Vaccinium* species indicated a risky hydraulic strategy with low hydraulic efficiency and safety as well as stomata closure at low water potential [72]. Nevertheless, the high risk of embolism formation is probably balanced by xylem repair capacities.

Limited soil water can have strong effects on the development, activity, and duration of the source and sink organs of plants [73]. Bilberry shifts more of its resources towards vegetative rather than reproductive growth when soil moisture availability is limited [73]. Irrigation may potentially increase yield by increasing berry numbers.

Drought stress affects bilberry differently depending on the phenological stage. Leaves are more sensitive to drought stress at the mature stage, and drought stress accelerates senescence at this stage [74]. A future increase in the severity of drought during the growing season is expected to increase the physiological stress of bilberry [75].

### 4.4. Temperature

Soil warming stimulated soil N cycling and shrub growth and development in the short term (2–3 years) [76]. Soil warming enhanced mean shoot production by 62%, leaf production by 44%, and leaf area by 46% during a 5-year experiment [76].

Soil and air warming advanced leaf development and stimulated growth in bilberry [76]. Plants produced 73% heavier shoots, 120% more stem biomass, and 52% more leaf biomass. Net N mineralization did not remain elevated in warmed plots for the entire 5-year study and appeared to decline in the last year. However, direct soil warming by 5 °C stimulates soil N cycling and shrub growth and development but does not alter plant community composition over the 5-year period [76].

September temperatures can be related to the general need for temperatures <10 °C for winter hardening and the accumulation of freeze protectants in *Vaccinium* spp. [59]. The frost hardiness of bilberry already starts to decrease significantly in December under temperatures elevated by 2–3 °C, with the deacclimation process accelerating throughout the winter [31,39]. Plants started to deharden and grow in mid-January after a fortnight’s exposure to +10 °C [39].

The winter extreme experiment (i.e., rapid snowmelt and exposure to unseasonable heat of +2–10 °C for some days) followed by a normal winter climate resulted in a 50% delayed bud burst of bilberry for 1 week and reduced flowering by more than 90% [39,77]. The damage is caused by winter warming events that are predicted to become more frequent under climate change.

Rixen et al. (2010) [12] found that the annual growth of bilberry was better at lower elevations than at higher ones but only in years with relatively cold summers. The climate may then affect plant species differently depending on the location of the population within the species distribution range. Similarly, a positive influence of the mean temperature from July to September on alpine, but not forest, berry yield was observed. Summer temperatures must be a limiting factor at the alpine site, where it improved annual bilberry performance throughout the study period.

These environmental factors—elevation, soil characteristics, nutrient availability, water supply, snow cover, and temperature—interact in complex ways to determine the growth, distribution, and success of bilberry in its various habitats. Understanding these interactions is crucial for predicting how this species may respond to changing environmental conditions in the future.

### 4.5. Nutrients

Nitrogen supply significantly alters growth patterns in bilberry, enhancing both root and shoot growth. Maximum plant dry weight, leaf production per plant, and mean leaf area increase by approximately 95%, 120%, and 75%, respectively, with no significant change in the specific leaf area (SLA) [47]. The main nitrogen storage organs in bilberry are roots and woody stems, accounting for 18% and 48% of nitrogen recovered in new shoots, respectively [47]. The first flush of growth relies mainly on nitrogen remobilization, contributing 80% of new leaf N [47]. The majority of nitrogen is allocated to roots and woody stems, including the rhizome, with maximum storage values reached in autumn [47]. Bilberry is more responsive to changes in nitrogen supply compared to other *Vaccinium* species and exhibits an opportunistic pattern of growth [47]. The rapid remobilization of nitrogen corresponds with this growth pattern, enabling the plant to develop independently of external nitrogen supply for a short time. This allows the plant to take advantage of habitats with temporal fluctuations in nitrogen availability [47].

The highest nitrogen additions (50 kg N ha^−1^ year^−1^) increased the mean leaf nitrogen concentration of understory shrubs. Within and across treatments, the amount of leaf chlorophyll a increased linearly with leaf nitrogen, implying an increase in the amount of light-harvesting compounds with leaf nitrogen [42]. Greater nitrogen availability likely contributed to the observed 62% increase in the current-year growth of bilberry, the dominant deciduous shrub in subarctic northern Sweden [76]. However, under warming, increased nitrogen mineralization rates alone will not increase nitrogen availability sufficiently to produce changes in community composition in subarctic tundra with low atmospheric inputs of nitrogen. Dramatic changes in community composition are more likely to occur where there are additional atmospheric nitrogen inputs [76].

Phosphorus is also known to have a positive effect on blueberry growth. It is essential for good root development and for seed and fruit formation. However, phosphorus is strongly adsorbed in mineral soil at a low pH [66,78]. The *Vaccinium* species show statistically significant Gaussian responses along soil nitrogen, phosphorus, and calcium gradients but not along other gradients, such as K and Mg [79].

Carbon and nitrogen concentrations and isotope compositions showed differences between stand types, which provide evidence that competition occurred between bilberry and its neighbors [22].

### 4.6. Snow

Increased temperatures are expected to lead to more severe drought regimes and large decreases in alpine snow amount and duration, especially below about 1500–2000 m elevation [80]. Increased snow depth exerts a considerable influence on tundra plant communities [81]. It has a large effect on the temporal pattern of the onset of the growing season, green-up, and flowering, delaying snowmelt by approximately 2 weeks in deep snow zones [81]. This effect is particularly pronounced in middle-height mountains [29].

Earlier snowmelt is predicted in many alpine ecosystems, and this abiotic change has critical implications for shrub community performance [82]. A longer growing season, driven by accelerated snowmelt, both fails to enhance growth and increases the risk of exposure to damaging spring freezing events at leaf maturity [82]. This suggests that the detrimental effects associated with an extended growing season may outweigh the benefits for some alpine shrub species. Spring frost was the cause of reduced growth and reduced flower production. However, advanced snowmelt will not decrease the cover of this species. Therefore, the structure and species dominance patterns in subalpine heath are not expected to change significantly in response to reduced snow cover [35].

Bilberry is generally regarded as a frost-sensitive species, but it is also able to recover vegetatively by producing new ramets after frost injury [62]. Increased shoot growth has been observed under experimental warming made by open-top (ITEX) chambers [83]. The results show that warming increases the cover of bilberry, which seems to enhance the nutrient sink strength of vegetation in the studied ecosystem [83]. There was a general decrease in shoot growth in mountain shrub species under earlier melt-out dates due to higher frost exposure [21]. Bilberry belongs to alpine species that delay development in response to postponed snowmelt [84,85]. The parameters of snow and phylogenetic constraints [84] interact, and the extension of the delay should be considered a species-specific response under genetic control [81].

## 5. Impact of Disturbances on Performance

### 5.1. Herbivory and Mowing

Dwarf shrubs are always present in extensively used hay meadows, and when hay-making is discontinued, they quickly spread [3]. The impact of herbivory by ungulates varies with altitude, having a moderate effect on heaths [65]. Moderate herbivory could promote the production of more branches and biomass in affected plants in the second year after damage compared to control plants. However, besides direct damage to flowers, fruits, and seeds during the current year, fruit production decreases in subsequent years. After shoot damage, new ramets emerged rapidly from dormant buds at the base of removed ramets [86]. Between 70 and 97% of the density relative to the control level was regained by the final harvest. However, only between 11 and 64% of the biomass relative to the control level was recovered. The survival and fecundity of the ramets were not affected by the disturbance, suggesting that bilberry exhibits moderate tolerance to typical levels of herbivory damage. The researchers of Tolvanen et al. (1994) [86] found that in the course of five growth seasons, bilberry stands recovered moderately after 25% and 50% harvesting, whereas recovery was poor after 100% damage. Clipping reduced branch growth in both new ramets and in new parts of unclipped ramets, with severe treatments decreasing growth more than light harvesting. Even though the establishment of new ramets was rapid, the slim growth of the ramets prevented the stands from recovering totally. Vowles and Björk (2019) [6] also documented the exclusion of large herbivores amplified deciduous tall shrub expansion. Herbivore pressure and stand thinning with more light can induce an increase in the accumulation of secondary metabolites [87]. Contrary to initial proposals, Pato et al. (2016) [88] suggested that *Vaccinium* does not always benefit from the cutting of *Calluna* in heathlands, indicating that management of Cantabrian Mountain heathlands should consider maintaining *Calluna* to facilitate *Vaccinium* growth.

The study on population dynamics of bilberry in relation to grazing intensity by deer (*Cervus elaphus*) in Norway found that the population growth rate of bilberry increased with the decreasing intensity of grazing [89]. The effect of herbivory on population growth rate was most profound in relatively resource-rich places. The species’ efficient clonal propagation after major environmental disturbances may provide it with a competitive advantage in certain grazing-influenced systems.

The effect of the combination of four-year clipping and fertilization was tested [90]. They found that repeated clipping caused reductions in both the length and diameter of shoots of bilberry, while fertilization alone increased shoot diameter but not length. Fertilization and clipping resulted in chemical changes in plant tissue, including increased nitrogen and slightly reduced carbon content. Bilberry has been found to recover even from severe damage within a few years [90]. This is likely due to the reallocation of both carbohydrates and nitrogen either from belowground parts of the plant or from other ramets within the genet [91]. However, repeated clipping resulted in a decreased abundance of bilberry, and the rate of recovery did not increase with fertilization, indicating that recovery may be carbon limited rather than nitrogen limited, as is generally expected for boreal plants.

### 5.2. Frost

After frost disturbance, the species recovered vegetatively (in terms of density and biomass) with a vigorous production of new ramets and large shoots in damaged ramets [62]. However, recovery did not occur sexually (in terms of flower production). Summer frost was found to be slightly more detrimental than spring frost, but the difference was significant only in the percentage of flowering ramets [62].

The ability of bilberry to recover from sporadic frosts increases its potential to persist in a changing environment, where increasing temperatures lead to earlier snowmelt in spring and increase the risk of frost injuries in plants that overwinter under snow [62]. However, Taulavuori et al. (1997) [31] found that even small elevations in air temperature can accelerate dehardening in bilberry, concluding that climatic warming may entail a real risk of early dehardening and further frost damage for the species.

While bilberry shows considerable resilience to various disturbances through its clonal growth strategy and ability to reallocate resources, repeated or severe disturbances can have long-lasting impacts on its abundance, growth form, and reproductive success [92]. Current-year stems in summer could sense the stress experienced by the previous-year stems in winter as a “stress memory” [32]. Understanding these complex responses is crucial for predicting and managing bilberry populations in changing environments.

### 5.3. Burning

Fire intensity significantly affects the recovery of *Vaccinium* species. According to Schimmel and Granström (1996) [93], after fires that burnt only the moss layer, *V. myrtillus* and *V. vitis-idaea* recovered to a pre-fire state within 2–4 years. Colonization from seeds was better following relatively deep-burning fires. Bilberry does not form a long-lasting seed soil bank [94]. However, after fires consumed most of the organic soil layer, seed bank species were badly affected, whereas species with rapid dispersal showed progressively better establishment with an increasing depth of burn. This variation in burn depth can have long-lasting impacts on vegetation composition and bilberry ecesis. Marozas et al. (2007) [95] found that the abundance of dominant bilberry recovered 5 years after a fire, though differences in moss species composition persisted even after 11 years. These findings highlight the complex and long-term effects of fire on forest understory communities.

## 6. Climate Change

Climate change also significantly impacts bilberry performance. Climate warming and increased atmospheric nitrogen deposition may result in a higher productivity of tundra plant communities, potentially increasing the role of competition [96]. In these changing conditions, the identity of neighbor species may become more important for recruitment than species richness.

Winter climate change is a critical driver of temperate ecosystems, and even short-term fluctuations in winter temperature can induce long-term (>3 years) shifts in the species abundance distribution of alpine communities [97]. Soil freeze–thaw cycles can cause nitrogen flushes, temporarily increasing N availability and nitrate leaching. These short-term climatic events can have long-term implications for ecosystem structure, with community composition regulating alterations in plant community development and competitive balance caused by soil warming pulses. Winter icing events can accelerate spring leaf emergence in bilberry [92]. This effect may be linked to increased shoot mortality caused by ice treatment, which reduces the proportion of alive shoots. This damage potentially leads to a greater allocation of resources to the remaining live shoots, enabling them to complete bud burst sooner. The redirection of resource allocation resulting from ice encasement damage could lead to increased spring growth.

Spring and summer frosts usually damage apical stems, leading to the rapid growth of new shoots from dormant buds [62]. The autumnal increase in anthocyanins supports recent findings on their role in photoprotection at low but non-freezing temperatures [32,39].

As underlined Vowles and Björk (2019) [6], evidence of the dwarf deciduous shrub expansion under climate change is included in common studies on shrubification, together with tall and evergreen shrubs. This generalizing approach makes the quantification of bilberry spread problematic worldwide. In order to improve our projections of bilberry feedback to climate change, we recommend strictly distinguishing between plant traits of chamaephytes in future research.

## 7. Conclusions

Bilberry as a subalpine deciduous chamaephyte exhibits a competitor-stress tolerator strategy. It thrives in acidic, nutrient-poor soils across various habitats from forests to alpine grasslands. The species shows broad ecological tolerance, propagating effectively through underground rhizomes and demonstrating a phalanx growth form. While it may suffer from inbreeding depression, it compensates through clonal growth advantages. Seed germination rates are typically low in the field, but high rates observed above its current altitudinal distribution suggest the potential for upward movement under climate change.

**Figure 1:**
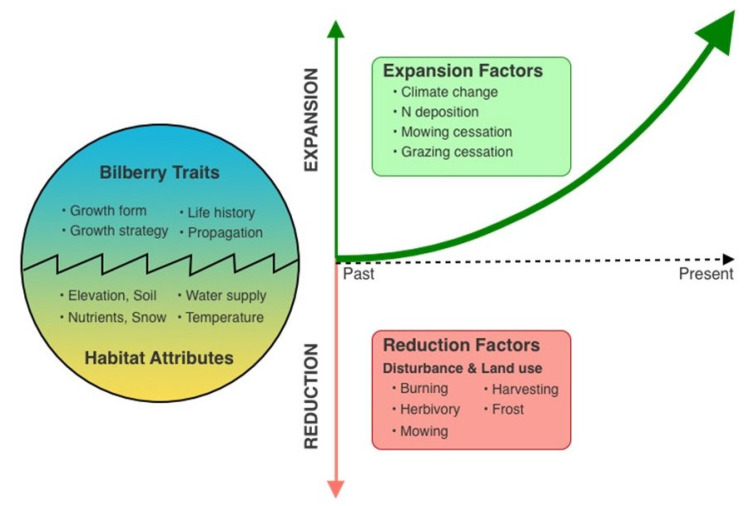
The essential controlling factors that affect the interaction of bilberry with its habitat and thus its expansion over time.

Bilberry’s growth and distribution are influenced by various environmental factors, including elevation, soil structure, pH, water availability, and nitrogen content. It adapts to different elevations by altering shoot size and leaf nitrogen distribution. Mycorrhizal associations play a crucial role in nutrient uptake in acidic soils. Despite its frost sensitivity, bilberry can regenerate vegetatively after frost injury. Overall, bilberry exhibits complex responses to changing environmental conditions, which has important implications for its future distribution and ecological role in alpine and subalpine ecosystems (Figure 1).

Bilberry significantly modifies the microclimate in mountain environments, reducing temperature and moisture fluctuations. The species facilitates plant litter accumulation and creates favorable conditions for specific animals and microorganisms. Moreover, its expansion influences soil microbial communities, shifting them towards ericoid mycorrhizal fungi and oligotrophic bacteria. This affects nutrient turnover, carbon cycling, and biodiversity in alpine ecosystems. Denser shrub cover leads to deeper snow, higher winter soil temperatures, and greater microbial activity, promoting shrub growth in the following summer.

Bilberry demonstrates complex responses to various ecological disturbances and environmental changes. It shows moderate tolerance to herbivory, with the ability to recover through the rapid emergence of new ramets, although severe damage can significantly reduce biomass and fruit production. Grazing intensity negatively affects bilberry population growth. The species exhibits resilience to clipping and fertilization, with recovery potentially being carbon limited rather than nitrogen limited. Bilberry can also recover vegetatively from frost disturbances, but repeated frost events may impact its abundance and reproductive success. Fire intensity significantly influences bilberry recovery, with varying effects based on burn depth.

Climate warming, along with reduced land management, has accelerated bilberry growth in treeline ecotones. The end of regular grazing and hay harvesting in most mountainous areas above the treeline, coupled with atmospheric nitrogen deposition and global temperature increase, has accelerated bilberry growth in the mountainous area. An increase in deciduous shrub species, including bilberry, is predicted due to lengthening growing seasons and enhanced nutrient availability. Future climate warming in tundra ecosystems is projected to lead to shrub-dominated tundra.

The management of bilberry expansion requires a nuanced approach, considering its resilience, historical land-use changes, and the complex interplay of environmental factors influencing its spread. The goal should be to limit, not eliminate, bilberry, as it is a natural part of subalpine communities. Soil properties in shrub removal treatments generally become more similar to grass treatments. However, the shrub legacy may persist longer after its removal compared to grass communities [69]. For grazing management, sheep and goats are suitable choices, as they nibble on woody plants. Grazing should be local (in fences) and extensive to prevent the destruction of bird nests or elimination of insects tied to specific food plants. Higher intensity grazing can lead to feces accumulation and subsequent eutrophication. Sensitive areas should be mowed to support the habitat mosaic even in the (sub)alpine belt.

Alpine plant communities do not preserve the condition that was created shortly after the end of the last Pleistocene glacial. Due to the extreme conditions, there are species and their communities that react very sensitively to changes in environmental conditions. Bilberry is a natural part of subalpine communities. The goal of management should be to limit it, not eliminate it. Management must be long-term, which increases its (not only) financial demands. Physically and technically, the removal of aboveground biomass from mountain terrain is challenging. Long-term comparative monitoring and experimental manipulation, including land use, should be rapidly developed.

## Data Availability

Data are contained within the article or Supplementary Materials.

## Supplementary Materials

The following supporting information can be downloaded at: https://www.mdpi.com/article/10.3390/plants13182633/s1, Table S1: The main sources of information used for the review about bilberry ecology, and basic conditions in which they were obtained.
