# Supplementary material for: Bilberry Expansion in the Changing Subalpine Belt
Source: Plants (Basel). 2024 Sep 20;13(18):2633. doi: 10.3390/plants13182633 (PMC11434830; doi:10.3390/plants13182633)
Supplement: Supplementary file 1 [file plants-13-02633-s001.zip › plants-3207043-supplementary.pdf]

Supporting information to the paper

Bilberry encroachment in the changing subalpine belt

Table S1. The main sources of information used for the review about bilberry ecology, and basic conditions in which they were obtained.

Reference	Year	Location	Habitat	Elevation (m a.s.l.)
Albert et al., 2004	2004	Belgium	heathland	550
Albert et al., 2008	2008	Belgium	heathland	550
Bokhorst et al., 2010	2010	Sweden (Abisko)	heathland	400
Boscutti et al., 2018	2018	Alps (Italy)	dwarf shrub communities (Rhododendro-Vaccinion)	2100
Broadbent et al., 2022	2022	Alps (Austria)	alpine grassland (Nardus stricta)	2200
Casolo et al., 2020	2020	Alps (Italy)	dwarf shrub communities (Rhododendro-Vaccinion)	2100
Collins et al., 2020	2020	global	alpine sites	
Coudun & Gégout, 2007	2007	France	forest	
Fernández-Calvo & Obeso, 2004	2004	N Spain		750 - 2000
Frak & Ponge, 2002	2002	France		950 - 2150
Ganthaler & Mayr 2015	2015	Alps (Austria)		791
Gerdol et al. 2000	2000	Alps (Italy)		1600
Gerdol et al., 2013	2013	Apennines (Italy)	dwar shrub heath	1741
Grelet et al., 2001	2001	NE Scotland		400
Grytnes, 2000	2000	W Norway	dwarf-shrubs heathland	1700
Hartley et al., 1999	1999	N Sweden (Abisko)	shrub heath	380
Hédl et al., 2012	2012	High Sudetes	subalpine vegetation	1300
Hegland et al., 2010	2010	W Norway	pine forest	100
Hejcman et al., 2006	2006	High Sudetes	subalpine vegetation	1500
Hill & Vander Kloet, 2005	2005	artificial conditions		
Iversen et al., 2009	2009	N Norway	birch forest	100
Janke, 1970	1970	Front Range (Colorado)	subalpine forest	
Kreyling et al., 2012	2012	NW Sweden	Norway spruce forest	260
Kreyling et al., 2012	2012	Germany	garden	365

Llorens et al., 2015	2015	N Wales (U.K.)	heathland	490
Llorens et al., 2015	2015	N Wales (U.K.)	heathland	490
Manninen & Tolvanen, 2017	2017	Finland	forest	420
Marozas et al., 2007	2007	S Lithuania		
Marozas et al., 2007	2007	S Lithuania	mixed forest	200
Miina et al. 2009	2009	Finland		
Myers-Smith & Hik, 2018	2018	Europe, N America	tundra	
Nestby et al., 2011	2011	review		
Nestby et al., 2014.	2014	Norway	norway spruce forest	
Nielsen et al., 2007	2007	SE Norway	norway spruce forest	485
Ögren, 1996	1996	Sweden (Umeå)	norway spruce forest	100
Olsen et al., 2022	2022	C Norway	forest/tundra	
Palmroth et al., 2014	2014	N Sweden	norway spruce forest	200
Pato & Obeso, 2012a	2012	NW Spain	deciduous forest/ heaths (Calluna vulgaris)	300 - 2000
Pato & Obeso, 2012b	2012	NW Spain	deciduous forest/ heaths (Calluna vulgaris)	300 - 2000
Pietilä et al., 1990	1990	N Finland	spruce and pine forest	70
Preece et al., 2012	2012	Sweden (Abisko)		400
Pudas et al., 2008	2008	Finland (Lapland)	dry forest	300-100
Rinnan et al., 2009	2009	NW Finland	birch forest	600
Ritchie, 1956	1956	British Isles		
Rixen et al., 2008	2008	SE Switzerland (Davos)	subalpine meadows	1530
Rixen et al., 2010	2010	SE Switzerland (Davos)	treeline	2200
Roth et al., 2021	2021	SW Germany	forest communities	
Saarinen et al., 2016	2016	Finland	coniferous forest	130
Saccone et al., 2017	2017	Finland (Lapland)		600
Selås et al., 2015	2015	S Norway	pine forest	250
Schimmel & Granström, 1996	1996	NE Sweden	norway spruce forest	300
Tahkokorpi et al., 2007	2007	N Finland	norway spruce forest	60
Taulavuori et al., 1997	1997	N Finland	norway spruce forest	60
Taulavuori et al., 2010	2010	N Finland	norway spruce forest	60

Theurillat & Guisan, 2001	2001	Europe (Alps)		
Timoshok, 2000	2000	West Siberian Plain		
Tolvanen et al., 1994	1994	N Finland	heath forest	250
Tolvanen, 1997	1997	N Finland	heath forest	260
Wheeler et al., 2014	2014	C Alps (Switzerland)	heathland	2300
Wipf et al., 2009	2009	C Alps (Switzerland)	heathland	2200
Woodward, 1986	1986	C Scotland		200 - 1100
